# Drought stress and re-watering affect the abundance of TIP aquaporin transcripts in barley

**DOI:** 10.1371/journal.pone.0226423

**Published:** 2019-12-17

**Authors:** Marzena Małgorzata Kurowska, Klaudia Wiecha, Katarzyna Gajek, Iwona Szarejko

**Affiliations:** Department of Genetics, Faculty of Biology and Environmental Protection, University of Silesia, Katowice, Poland; Birla Institute of Technology and Science, INDIA

## Abstract

Tonoplast Intrinsic Proteins (TIP) are plant aquaporins that are primarily localized in the tonoplast and play a role in the bidirectional flux of water and other substrates across a membrane. In barley, eleven members of the *HvTIP* gene subfamily have been identified. Here, we describe the transcription profile of the *HvTIP* genes in the leaves of barley seedlings being grown under optimal moisture conditions, drought stress and a re-watering phase. The applied drought stress caused a 55% decrease in the relative water content (RWC) in seedlings, while re-watering increased the RWC to 90% of the control. Our analysis showed that all *HvTIP* genes, except *HvTIP3;2*, *HvTIP4;3* and *HvTIP5*.*1*, were expressed in leaves of ten-day-old barley seedlings under optimal water conditions with the transcripts of *HvTIP2;3*, *HvTIP1;2* and *HvTIP1;1* being the most abundant. We showed, for the first time in barley, a significant variation in the transcriptional activity between the analysed genes under drought stress. After drought treatment, five *HvTIP* genes, which are engaged in water transport, were down-regulated to varying degrees, while two, *HvTIP3;1* and *HvTIP4;1*, were up-regulated. The HvTIP3;1 isoform, which is postulated as transporting hydrogen peroxide, expressed the highest increase of activity (ca. 5000x) under drought stress, thus indicating its importance in the response to this stress. Re-hydration caused the return of the expression of many genes to the level that was observed under optimal moisture conditions or, at least, a change in this direction Additionally, we examined the promotor regions of *HvTIP* and detected the presence of the *cis*-regulatory elements that are connected with the hormone and stress responses in all of the genes. Overall, our results suggest that 7 of 11 studied *HvTIP (HvTIP1;1*, *HvTIP1;2*, *HvTIP2;1*, *HvTIP2;2*, *HvTIP2;3*, *HvTIP3;1*, *HvTIP4;1)* have an important function during the adaptation of barley to drought stress conditions. We discuss the identified drought-responsive *HvTIP* in terms of their function in the adaptation of barley to this stress.

## Introduction

Aquaporins (AQP) are membrane intrinsic proteins (MIP) that accelerate the passive movement of water and other substrates across the membranes in organisms such as Archaea, Eubacteria and Eucaryota including fungi, plants and animals [1–4]. In addition to water, some major intrinsic protein (MIP) family members can also transport glycerol, CO_2_, urea, ammonia, hydrogen peroxide, boron, silicon, arsenite, antimonite, lactic acid and O_2_ [5]. The molecular weight of the AQP family members ranges from 23 to 31 kDa [5].

Depending on the membrane location and amino acid sequence, the higher plant MIP, including AQP, are typically divided into five subfamilies: the plasma membrane intrinsic proteins (PIP), tonoplast intrinsic proteins (TIP), nodulin-26-like proteins (NIP), small, basic intrinsic proteins (SIP) and the uncategorised X intrinsic proteins (XIP) [5]. According to their molecular structure, all MIP consist of: six transmembrane helices, five inter-helical loops, two short helices that contain the highly conserved Asn-Pro-Ala (NPA) motif that forms the pore and the so-called aromatic/arginine (ar/R) selectivity filter, including four amino acids that act as a size-exclusion barrier because they form the narrowest part of a pore [5, 6, 7]. The NPA motif not only plays a role in regulating membrane transport but also in protein location [8]. Four AQP monomers assemble to form a tetrameric holoprotein [5].

The tonoplast intrinsic proteins (TIP) that are located in the tonoplast facilitate the rapid osmotic equilibration between a vacuole and a cytosol [9]. A genome-wide analysis conducted in 34 species (both monocots and dicots) by Bezzera-Neto and coworkers [4] showed that the number of *TIP* genes ranged from six in moso bamboo (*Phyllostachys edulis*) [10] to 35 in canola (*Brassica napus*) [11]. In barley (*Hordeum vulgare*), the *TIP* subfamily comprises 11 members [12].

In barley, several past studies have shown that *HvTIP1;1*, *HvTIP1;2*, *HvTIP2;1*, *HvTIP2;2*, *HvTIP2;3*, *HvTIP3;1*, *HvTIP4;1* and *HvTIP5;1* are expressed in different patterns during leaf development [13]. *HvTIP1;1* facilitates the water uptake in roots [14]. Abiotic stress (salt, heavy metals and nutrient deficiency), treatment with abscisic acid (ABA) and gibberellic acid (GA) modulate the expression of some members of the *TIP* subfamily in roots and shoots, e.g., *HvTIP1;2*, *HvTIP2;1*, *HvTIP2;2*, *HvTIP2;3* and *HvTIP4;1* [15]. To the best of our knowledge, no research has been carried out on the impact of drought stress on the *HvTIP* expression in barley. Although there are such reports for other plant species, namely *Arabidopsis thaliana*, *Festuca arundinacea* and *Nicotiana glauca*, they only focused on some members of the *TIP* subfamily [16, 17, 18, 19].

Changes in the expression level of the *TIP* genes under water shortages indicate their involvement in response to such conditions *in planta*. Other indications on the function of specific aquaporins may be found using functionality tests. In barley, it was found that three aquaporins from the TIP subfamily, HvTIP1;1, HvTIP1;2 and HvTIP2;3, are able to transport water [13]. However, this ability was not confirmed for HvTIP2;3 in a different study [15]. In addition to the expression studies and functionality tests, a prediction of the transported substrates using bioinformatics tools might be helpful in determining the role of specific aquaporins. Based on the key structural features of the amino acid sequences of HvTIP, the transport of substrates other than water was predicted [20]. This analysis suggested that, HvTIP1;1 and HvTIP1;2 may be involved in the transport of H_2_O_2_ and urea; HvTIP2;1, HvTIP2;2 and HvTIP2;3 in the transport of ammonia, formamide and H_2_O_2_; HvTIP3;1 and HvTIP3;2 in the transport of H_2_O_2_ only and HvTIP4;3 in the transport of glycerol and urea, while such substrates have not been determined for HvTIP4;1, HvTIP4;3 and HvTIP5;1 [12]. Interestingly, the potential to transport the signalling molecules H_2_O_2_ and ammonia was restricted to only some TIP in barley, while the potential to transport urea and glycerol was widespread among the members of diverse subfamilies of MIP [12]. The open question is whether the aquaporins that are permeable to substrates other than water still retain the ability to transport water [20].

In the present study, we investigated the expression of the barley tonoplast aquaporin genes (*HvTIP*) in response to drought stress. We determined the transcription profile of 11 *HvTIP* genes in the leaves of ten-day-old barley seedlings being grown under optimal moisture conditions, drought stress and after a re-watering phase. Additionally, we examined the promotor regions of *HvTIP* for the presence of the *cis*-regulatory elements that are connected with the hormone and stress responses. The analysis revealed a significant variation in transcriptional activity between the analysed genes under different environmental conditions. The identified drought-responsive *HvTIP* are discussed in terms of their function.

## Materials and methods

### Plant material

The two-row spring barley variety ‘Sebastian’ was used in the study. This cultivar is characterised by its high yield potential, high tillering, good malting quality, resistance to lodging, high resistance to both stem rust (*Puccinia graminis*) and leaf rust (*Puccinia hordei*) and moderate resistance to powdery mildew (*Blumeria graminis* f.sp. *hordei*), net blotch (*Pyrenophora teres*) and scald (*Rynchosporium secalis*) [21].

### *In silico* analysis of the barley *TIP* genes (*HvTIP*)

The members of the *TIP* gene subfamily in barley are already known [12]. The sequences that are available in GenBank (http://www.ncbi.nlm.nih.gov/genbank/) or in PLAZA 3.0 Monocots—Hordeum vulgare (https://bioinformatics.psb.ugent.be/plaza/versions/plaza_v3_monocots/organism/view/Hordeum+vulgare) were used in the BLAST searches using the Ensemble Plant Databases–Hordeum vulgare subsp. vulgare Ensemble Genomes 43 (http://plants.ensembl.org/Hordeum_vulgare/Info/Index) for the analyses and the HORVU number identification, chromosome location and splice variants. To identify the putative stress-responsive *cis*-elements in the promoter regions of the *HvTIP* genes, one kb of the upstream sequences relative to the transcription start sites were searched against the PlantCARE (Plant Cis-Acting Regulatory Elements, http://bioinformatics.psb.ugent.be/webtools/plantcare/html/) [22]. A gene similarity tree of the barley aquaporins from the TIP subfamily was constructed using Clustal Omega (https://www.ebi.ac.uk/Tools/msa/clustalo) software using the Neighbour Joining Clustering method. A multiple sequence alignment of the partial amino acid sequences of HvTIP in barley was performed using Clustal Omega software. The conserved NPA motifs and ar/R selectivity filters were determined by a careful visual inspection of the multiple sequence alignment of 11 amino acid sequences of HvTIP, which were previously characterised [12].

### Drought stress treatment and Relative Water Content (RWC) analysis

**Fig 1:**
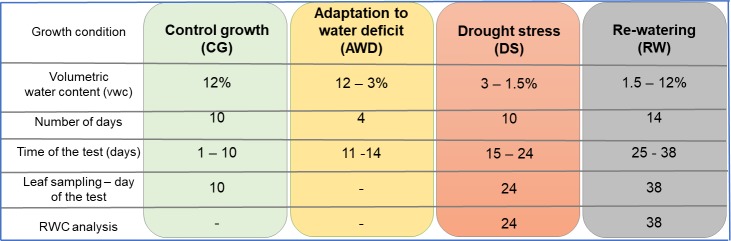
Drought stress treatment during seedling stage for the ‘Sebastian’ variety. The plant material was collected for RNA isolation after: (1) 10 days of growth under optimal water condition, soil moisture 12% vwc–**c**ontrol **g**rowth (CG); (2) 14 days of drought stress including 4 days of **a**daptation to **w**ater **d**eficit (AWD), soil moisture gradual decrease from 12% to 3% and 10 days of **d**rought **s**tress (DS), soil moisture 3% to 1.5%; (3) after 14 days of **r**e-**w**atering (RW), soil moisture gradual increased from 1.5% to 12%. The RWC analysis was performed on 10 **d**ays **a**fter **s**owing (DAS) under control growth (CG), 25 DAS under drought stress and on 38 DAS under re-watering.

The barley seeds were sown in Petri dishes containing water-soaked vermiculite and kept at 4°C in darkness for two days. Then, the Petri dishes were transferred into a greenhouse (where the experiment was performed) for another two days. On the 4^th^ day, the germinated seedlings were transferred into pots (400 x 140 x 175 mm) and filled with soil, which was prepared as described elsewhere [23–26]. Duration of the growth of seedlings for physiological and molecular analysis is given as **d**ays **a**fter **s**owing (DAS) in the manuscript. Fifteen seedlings were placed into each pot. At the time of plantlet transfer, the soil moisture was 12% vwc (volumetric water content) in all of the pots. During the entire experiment, the soil moisture was monitored on a daily basis using a Time-domain reflectometer (TDR) EasyTest (Institute of Agrophysics, Polish Academy of Sciences, Poland). The drought assay that was used includes four phases: **c**ontrol **g**rowth (CG) at 12% vwc for ten days from placing the seedlings into the pots; **a**daptation of the plants to a **w**ater **d**eficit (AWD)–four days of gradually decreasing the soil moisture from 12% to 3% vwc; the **d**rought **s**tress (DS) at 3%-1.5% vwc for ten days and the **r**e-**w**atering phase (RW) at 12% vwc for 14 days (Fig 1).

The entire drought test lasted 38 days. The seedlings were grown in a greenhouse for the CG, AWD and RW phases at 20°C/18°C with a 16/8 h photoperiod and 400 μEm^-2^s^-1^ light intensity, which was provided by fluorescent lamps. For the drought stress (DS) treatment, the seedlings were transferred into a growth chamber, where the temperature regime was set to 25°C/20°C with a 16/8 h photoperiod with the same light intensity as in the greenhouse. At three time points: (1) after ten days of growth in the control conditions (10 **d**ays **a**fter **s**owing, DAS); (2) after 14 days of drought including the AWD and DS phases (24 DAS) and (3) after 14 days of rehydration (38 DAS), the leaf material was collected to isolate the RNA and perform the molecular study (Fig 1).

The water loss rates of the detached leaves were measured by monitoring the fresh weight loss at the indicated time points (Fig 1). RWC was calculated based on the formula: RWC = (F_w_-D_w_) / (T_w_-D_w_) x 100%, where: F_w_ is the fresh weight of the detached second leaf, T_w_ is the turgid weight of the second leaf that has been incubated in distilled water for 24 h in darkness after detachment and D_w_ is the dry weight of the second leaf that has been dried in a dryer at 60°C for 48 h. The analysis was performed in three biological replications, using three plants per replication.

### *HvTIP* expression analyses

The relative expression of the *HvTIP* genes was assessed using quantitative real-time RT-PCR (RT-qPCR). The total RNA was extracted from each barley sample using a TriPure Isolation Reagent according to the manufacturer’s protocol (Roche Life Science), which is based on the method of Chomczynski [27]. Before reverse transcription, one microgram (μg) of the total RNA was treated with RNase-free DNase I (Fermentas) for 30 min to degrade any residual genomic DNA. Next, a RevertAid First Strand cDNA Synthesis Kit (Thermo Scientific) was used to synthesise the first-strand cDNA. The cDNA that was obtained was then diluted 1:5 with ddH_2_O and used as the template for the quantitative PCR. The 10 μl qPCR reaction mix contained 2,5 μl of diluted cDNA, 1 μl of the primer pair mixture (5 μM) and 5 μl of 2 × Master Mix (LightCycler 480 SYBR Green I Master; Roche). The RT-qPCR reactions were performed at 95°C for 5 min followed by 45 cycles of 95°C for 10 s, 58°C for 20 s and 72°C for 10 s. The value of the relative expression level was normalised to a reference gene *ADP* (*ADP-ribozylation factor 1*, accession no. AJ508228.2), which is suitable for studying drought-induced changes in the gene expression at the seedling stage in barley [28]. The transcript level of the *HvTIP* in the leaves of ten-day-old barley seedlings under optimal growth conditions was calculated using the formula: Ct target gene–Ct reference gene. To analyse the expression under drought stress, the relative expression of each *HvTIP* at a given time point was determined as the fold change of its expression under the treatment conditions relative to its expression under the control conditions according to the delta-delta Ct method [29]. Three biological replications were used to analyse the gene expression with a sample of one seedling per replication. Each sample was analysed in two technical replicates. The relative expression data were analysed using the LinReq software tool [30], Statistica (13.1; Dell) and the one-way ANOVA followed by Fisher Least Significant Difference (LSD), differences with *p*-values <0.05 were considered to be statistically significant.

## Results

### Analysis of the *HvTIP* promoter reveals the presence of stress- and hormone-related elements

**Fig 2:**
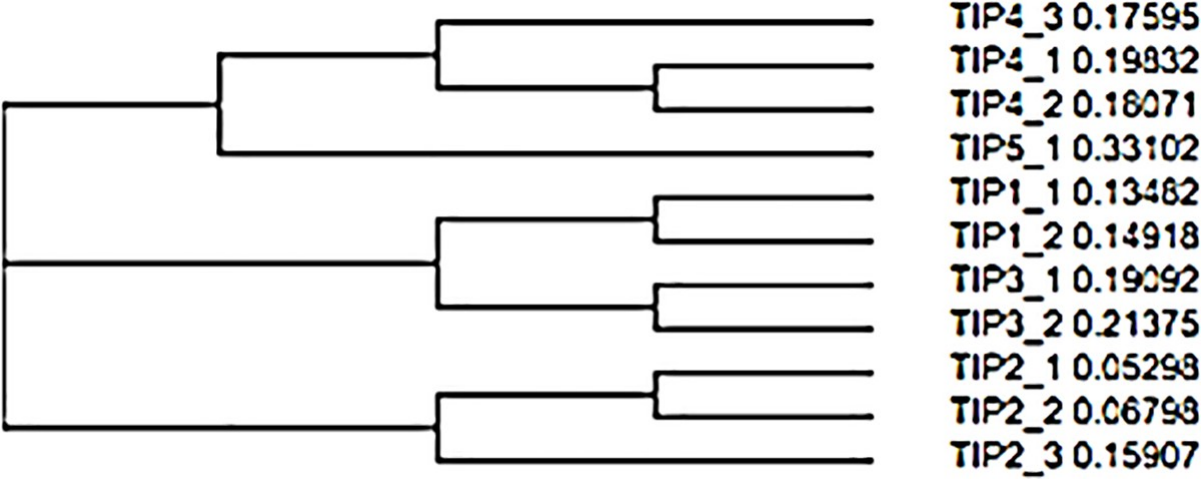
Tree of genetic similarity of barley aquaporins form TIP subfamily based on amino acid sequences of *HvTIPs* using Clustal Omega software with the Neighbour-Joining Clustering method.

The EnsemblePlants database was used to identify the promotor sequences of the *HvTIP* genes and the mRNA sequences that are deposited in the NCBI database were used as the query. The *HvTIP* genes in the barley genome were previously identified by Hove and coworkers [12]. A genetic similarity tree was created based on the amino acid sequences of the barley aquaporins from the TIP subfamily using the Neighbour Joining Clustering method. The three main clusters are shown: a): HvTIP4;3, HvTIP4;1, HvTIP4;2 and HvTIP5;1, b): HvTIP1;1, HvTIP1;2, HvTIP3;1 and HvTIP3;2 and c): HvTIP2;1, HvTIP2;2 and HvTIP2;3, which are further subdivided into smaller groups in which the isoforms that have the same main number occur (Fig 2).

The *HvTIP* genes are located in all barley chromosomes except for 5H (Table 1). The number of splice variants for individual genes that were found in EnsemblePlants ranged from 2 to 18. These data are provided by the International Barley Genome Sequencing Consortium and are based on a deep RNA sequencing project (RNA-seq) [31]. The abundant amount of alternative splicing for some of the *HvTIP* genes suggest that post-transcriptional processing might be their important regulatory mechanism. There was no high confident hit for *HvTIP5;1* during the BLAST analysis using an amino acid sequence as the query in the Ensemble database (Table 1).

**Table 1:** Characteristics of *HvTIP* genes.

HvTIP genes	Number in database			Chromosomal location	No. of splice variants
	Ensemble	NCBI*	PLAZA*		
1;1	HORVU4Hr1G079230	AB540221	MLOC_73301	4H	5
1;2	HORVU3Hr1G116790	AB540226	MLOC_58872	3H	13
2;1	HORVU6Hr1G062980	AB540222	MLOC_66094	6H	2
2;2	HORVU2Hr1G097780	AB540223	-	2H	18
2;3	HORVU7Hr1G081770	AB540224	MLOC_22808	7H	10
3;1	HORVU1Hr1G043890	AB540228	MLOC_51183	1H	5
3;2	HORVU0Hr1G005250	AK373620	MLOC_72436	chrUn	3
4;1	HORVU4Hr1G085250	AB540225	MLOC_71237	4H	7
4;2	HORVU3Hr1G031680	-	MLOC_71267	3H	9
4;3	HORVU3Hr1G031620	-	MLOC_69640	3H	5
5;1	not determined	AB540227	-	-	-

*based on Hove et al. 2015

**Fig 3:**
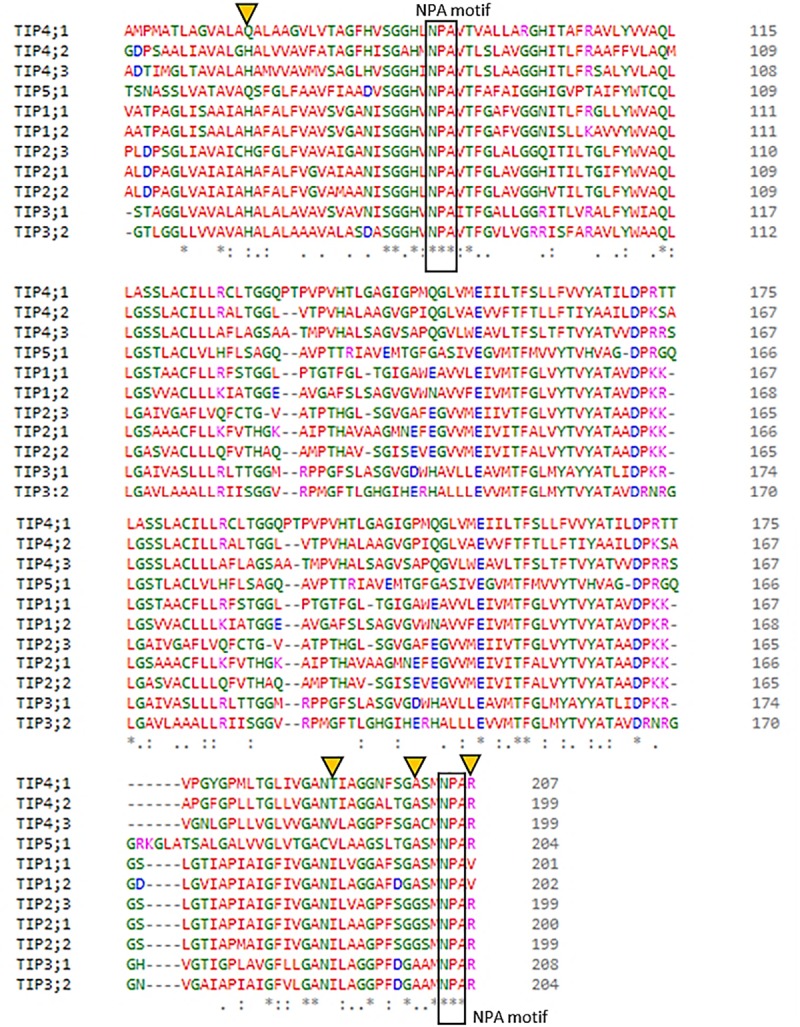
Multiple sequence alignment of partial amino acid sequences of HvTIPs in barley. NPA motifs and ar/R selectivity filters were labeled with rectangular boxes and yellow triangles, respectively.

The multiple sequence alignment of the partial amino acid sequences of the HvTIP in barley showed the presence of both conserved and specific motifs. The dual NPA (Asparagine-Proline-Alanine) motifs were conserved among all 11 HvTIP. However, the ar/R (aromatic/Arginine) selective filters, which consist of four residues, were highly variable and contained the following amino-acids at these positions: [H/Q]–[I/T/V]–[A/G]–[V/R] (Fig 3).

To identify the putative stress-responsive *cis*-elements in the promoter regions of the *HvTIP* genes, one kb of the upstream sequences relative to the transcription start sites were searched against the PlantCARE database. The presence of several putative *cis*-regulatory elements that are mainly connected with abiotic stress (Table 2) and phytohormone (Table 3) responses were revealed. A total of four types of stress-related *cis*-elements were detected, including LTR (low temperature-responsive elements), DRE (dehydration-responsive element), TC-rich repeats (defense and stress-responsive elements) and MBS (the MYB binding site that is involved in drought-inducibility). Additionally, the *cis*-elements that are recognised by transcription factors (TF) from the MYB and MYC family, which may play a role in the response to abiotic stress were detected in the promoters of *HvTIP* (Table 2). Furthermore, four types of elements that possibly participate in the response to hormones, such as abscisic acid (ABA), methyl jasmonate (MeJA), gibberellins (GA) and auxin, were also identified (Table 3).

**Table 2:** Stress-related *cis*-acting elements found in the *HvTIP* promoters.

Motif	Sequence	Function of cis-acting element	*HvTIP* genes
CCAAT-box	CAACGG	MYBHv1 binding site	1;1,
DRE-core	GCCGAC	Dehydration responsive	1;1, 2;2, 2;3, 4;1, 4;3, 5;1
MBS	CAACTG	MYB binding site involved in drought-inducibility	2;1, 2;2, 3;2, 5;1
MYB	CAACCA	Recognized by TF from MYB family in response to stress	1;2, 2;2, 4;1, 5;1
	CAACAG		2;1, 3;1, 4;2,
	TAACCA		1;2, 2;1, 2;2, 5;1
	TAACTG		1;1, 1;2, 2;2, 4;2, 5;1
	CCGTTG		1;1, 4;2
MYC	CAATTG	Recognized by TF from MYC family in response to stress	2;2, 5;1
	CATTG		1;1, 2;1, 4;3
	CATTTG		2;3, 4;1, 4;2,
	CATGTG		4;1, 4;2,
	CATGTC		2;3,
	TCTCTTA		2;1
LTR	CCGAAA	Low-temperature responsive	1;1, 4;1, 4;2
TC-rich repeats	GTTTTCTTAC	Defense and stress responsive	4;2,

TF- transcription factor

All of the 11 genes contained at least one *cis*-element that is connected with the stress or hormone response. The best represented were two regulatory elements: the TGACG-motif and CGTCA-motif, which were found in all of the promoters that were investigated. These elements are implicated in plant responses to methyl jasmonate (MeJA), a well-known primary signal in plant defense and stress response. All of the *HvTIP* promoter regions, except for *HvTIP2;1*, contained at least one ABA-responsive element (ABRE), which is involved in the ABA response and in ABA-mediated abiotic stress signalling. Conversely, only some of the *HvTIP* promoters contained an auxin- or gibberellin-responsive element (Table 3). Other well-represented elements were: DRE (dehydration responsive), which was detected in 6 of the 11 *HvTIP* promoters and MBS (a MYB binding site that is involved in drought-inducibility), which was found in 4 of the 11 promoters. The functionality of the predicted *cis*-elements requires experimental validation.

**Table 3:** Hormone-related *cis*-acting elements in the *HvTIP* promoters.

Motif	Sequence	Function of *cis*-acting element	*HvTIP* genes
ABRE	ACGTG	Abscisic acid responsive	1;1, 2;2, 2;3, 3;1, 3;2, 4;1, 4;2, 4;3, 5;1
	CACGTG		2;2, 3;1, 3;2, 5,1
	TACGGTG		1;2
	AACCCGG		4;1
	GACACGTGGC		1;1, 1;2, 3;2
	CGCACGTGTC		3;1, 3;2
	CGTACGTGCA		2;2, 4;2, 5;1
	GCAACGTGTC		4;2
	GCCGCGTGGC		4;3
ABRE3a	CACGTA		3;1
	TACGTG		4;2, 4;3
ABRE4	TACGTG		3;1
	CACGTA		4;2, 4;3
Auxx-core	GGTCCAT	Auxin-responsive	1;1,
TGA-element	AACGAC		1;1, 2;1, 4;1, 4;2
GARE-element	TCTGTTG	Gibberellin-responsive	2;1, 3;1, 4;2
P-box	CCTTTG		1;2
TACT-box	TATCCCA		3;1
CGTCA-motif	CGTCA	Methyl jasmonate-responsive	1;1, 1;2, 2;2, 2;3, 3;1, 3;2, 4;1, 4;2, 4;3, 5;1
TGACG-motif	TGACG		1;1, 1;2, 2;2, 2;3, 3;1, 3;2, 4;1, 4;2, 4;3, 5;1

### Relative Water Content (RWC) analysis showed the severity of drought stress assay that was used

Fifteen-day-old seedlings of the ‘Sebastian’ variety were subjected to severe drought stress treatment that was preceded by a four-day period of gradual decrease in the amount of water (Fig 1). After the drought treatment, the plants were re-watered for 14 days. Control seedlings of the ‘Sebastian’ variety were grown simultaneously under optimal water conditions.

**Fig 4:**
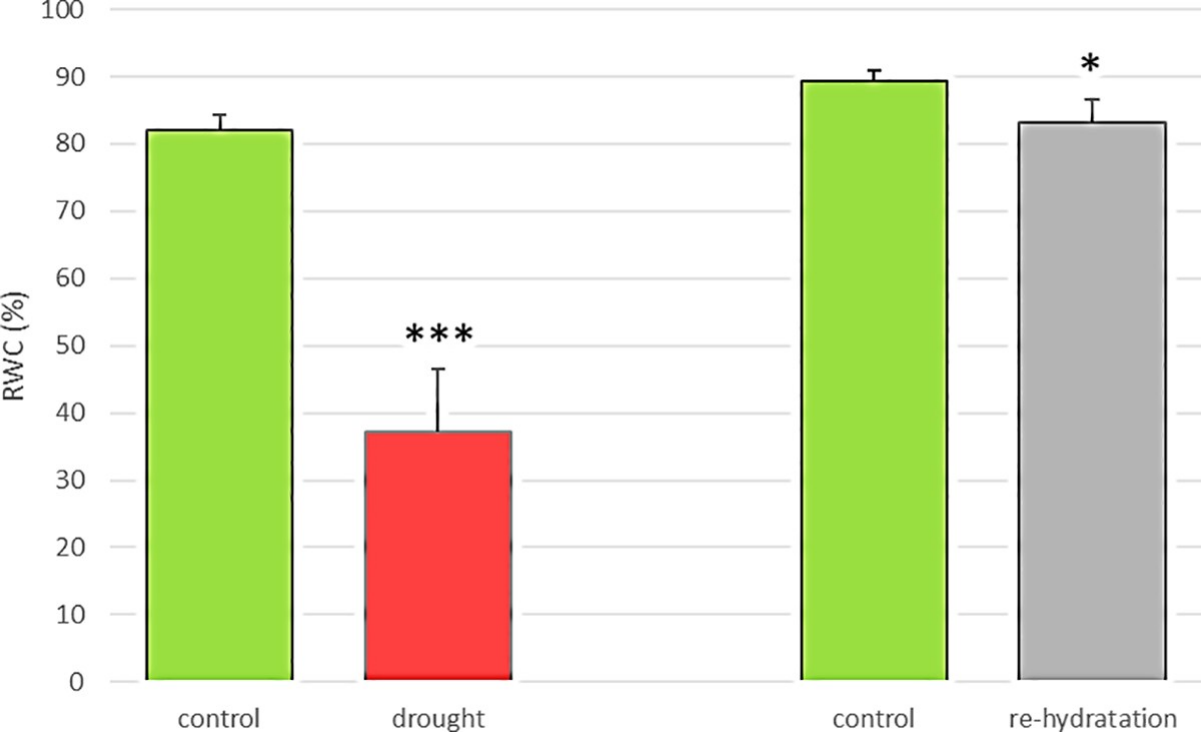
RWC measured in the second leaf of ‘Sebastian’ variety twice during seedlings growth: On 24^th^ day of growth under control growth (CG) and after 14 days of drought stress (including 4 days of adaptation to water deficit and 10 days of severe drought), on 38^th^ day of growth under CG and after 14 days of re-watering (RW). Data are means of three replicates per treatment combination. The statistical analysis was performed using the one-way ANOVA followed by Fisher Least Significant Difference (LSD) test (*P < 0.05; ***P < 0.001) to assess the differences between control growth (CG) and different growth conditions.

**Fig 5:**
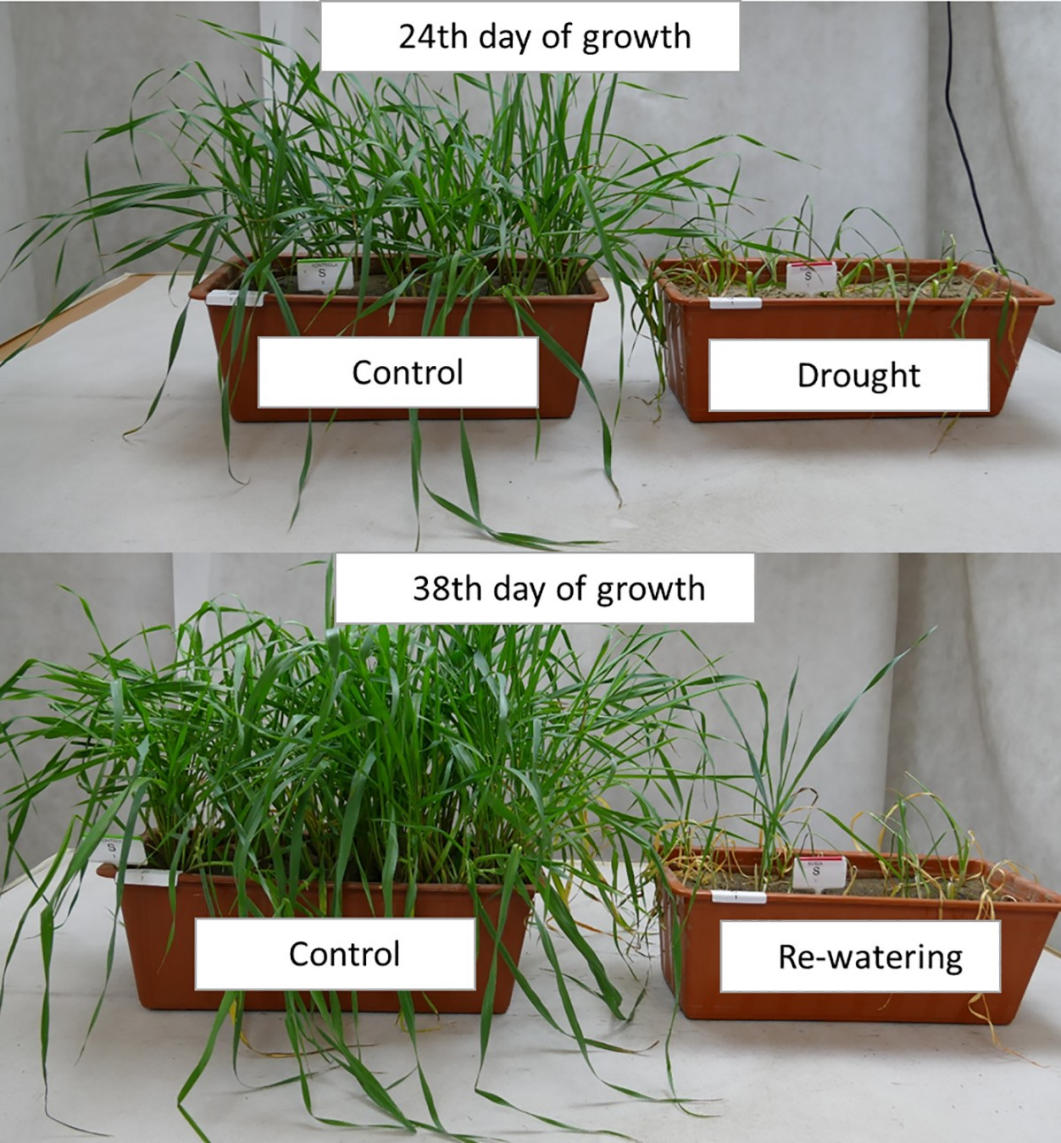
Phenotypes of ‘Sebastian’ seedlings during drought assay under control growth (CG) and after 14 days of drought stress (DS) on 24^th^ DAS; under CG and after 14 days of re-watering (RW) on 38^th^ DAS.

The drought stress that was applied caused a significant decrease in the relative water content (RWC) in the ‘Sebastian’ seedlings. The RWC of the drought-treated seedlings only reached 45% of the RWC of the control plants (Fig 4). During the 14-day period of re-watering, the seedlings began to grow again and their RWC increased to 93% of the control plants at the end of this phase (Figs 4 and 5).

### Changes in the *HvTIP* expression profiles after drought stress and re-watering

**Fig 6:**
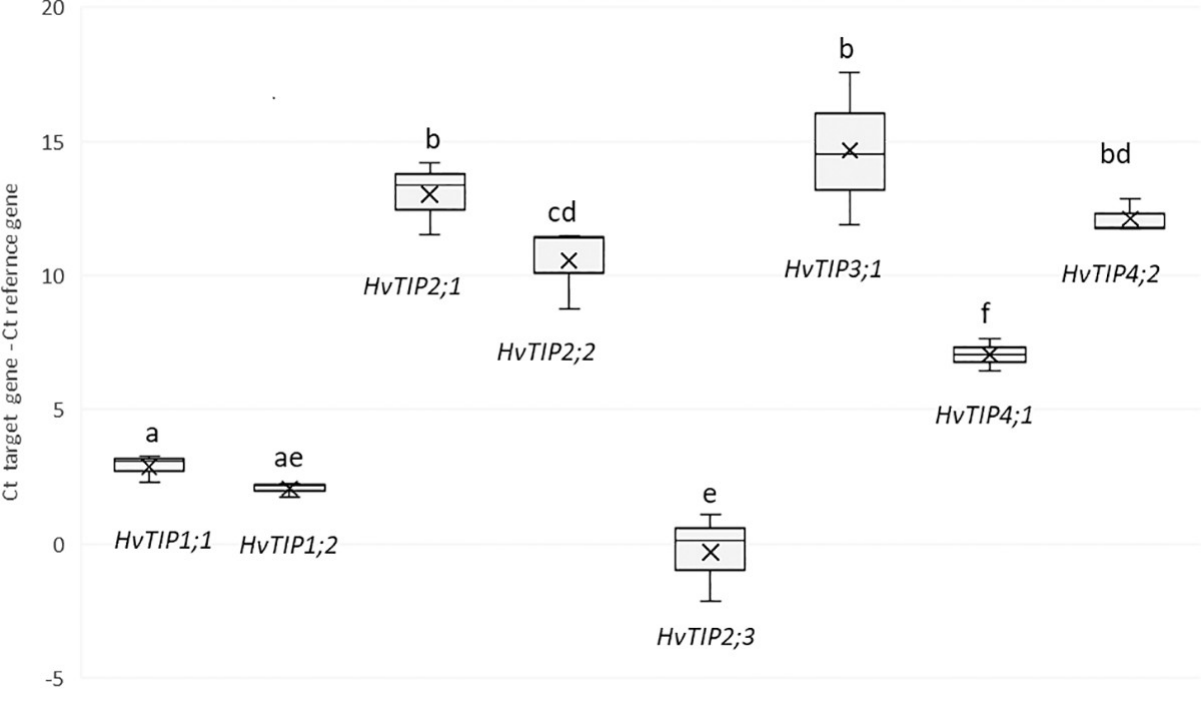
Abundance of transcript level of *HvTIPs* under control growth (CG) in the leaves of 10-days old barley seedlings calculated from formula: Cycle threshold (Ct) target gene–Ct reference gene (*ADP*, *ADP-ribozylation factor 1*). The box-and-whisker plot shows the minimum and maximum (whisker) and median (cross) of a set of data. The statistical analysis was performed using the one-way ANOVA followed by Fisher Least Significant Difference (LSD) test to assess the differences between different expression level of *HvTIPs*. Statistically significant differences (P < 0.05) are marked by different letters. All samples were analyzed in triplicate.

We first assessed the number of *HvTIP* transcripts in the seedlings that were grown in the optimal soil moisture conditions. Our analysis showed that most of the *HvTIP* genes, except for *HvTIP3;2*, *HvTIP4;3* and *HvTIP5*.*1*, were transcribed in the leaves of the ten-day-old barley seedlings. As is shown in the Fig 6, the most highly expressed genes were: *HvTIP2;3*, *HvTIP1;2* and *HvTIP1;1*, which could indicate they are indispensable during this stage of leaf development. Conversely, the *HvTIP3;1* gene was expressed at the lowest level, followed by the *HvTIP2;1* and *HvTIP2;2* genes. The expression level of *HvTIP3;1* was much lower and the expression level of *HvTIP2;*3 was slightly higher compared to the reference *ADP* gene encoding ADP-ribozylation factor 1.

**Fig 7:**
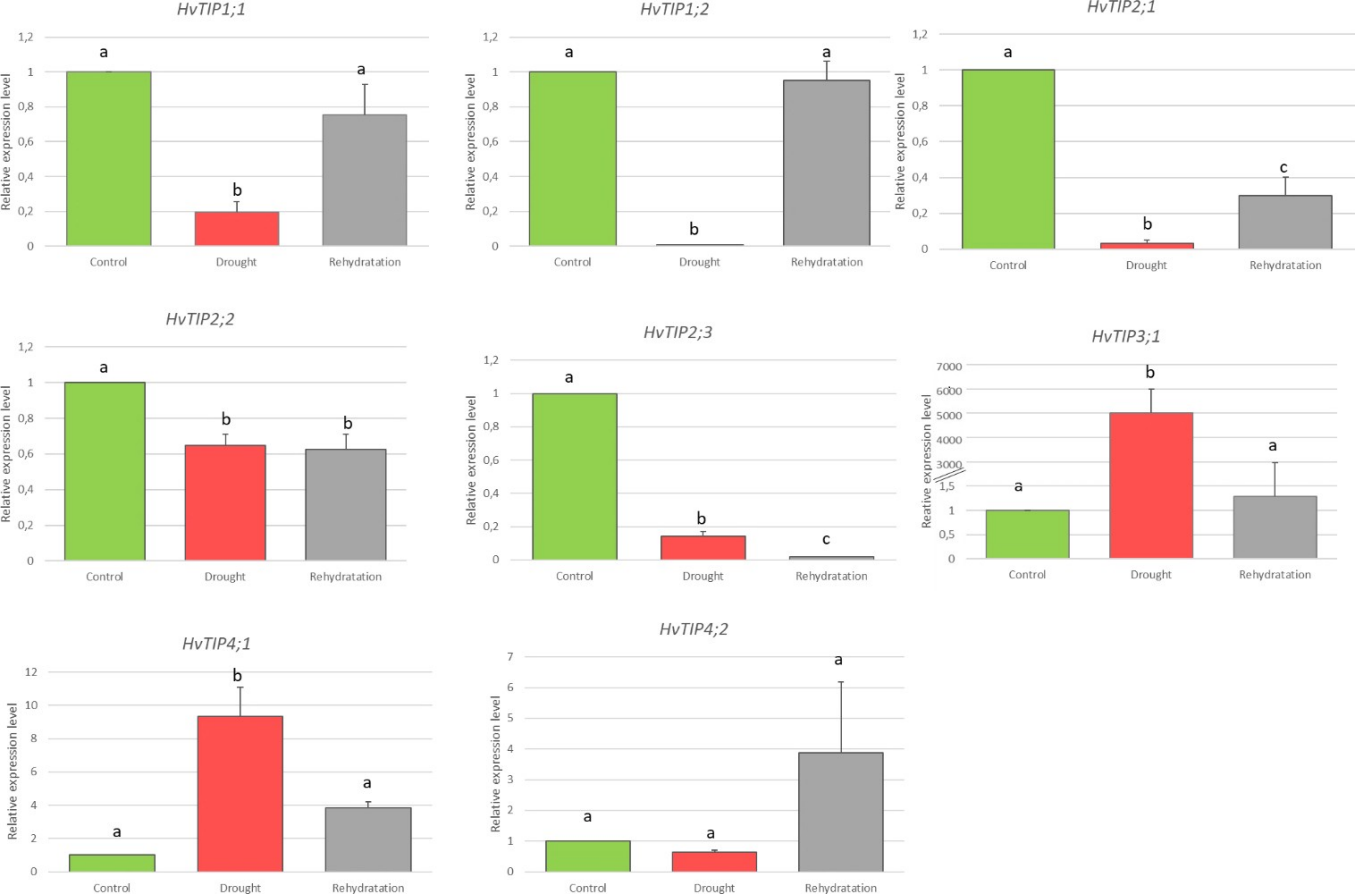
Expression patterns of *HvTIP* genes under drought stress and re-watering in barley leaves. Relative expression level was evaluated in comparison to control plants which were grown under optimal soil moisture. The statistical analysis was performed using the one-way ANOVA followed by Fisher Least Significant Difference (LSD). Statistically significant differences (P < 0.05) are marked by different letters.

Because all 11 *HvTIP* genes had the drought-responsive *cis*-elements in their promoters, we decided to evaluate their transcriptional activity in seedlings that were exposed to ten days of severe drought stress followed by 14 days of re-watering. Three genes: *HvTIP3;2*, *HvTIP4;3* and *HvTIP5*.*1*, which were not transcribed in the leaves of the ten-day-old seedlings under optimal moisture conditions, were also not induced by drought treatment. However, the majority of the *HvTIP* genes that were transcriptionally active in the control, had significant changes in their expression level in response to the drought stress that was applied and the return to the optimal water conditions. The timing and magnitude of the observed changes were diverse among the analysed genes. Based on their expression profiles, we distinguished two main groups of *HvTIP*: the first group of five genes that were down-regulated after drought stress (*HvTIP1;1*, *HvTIP1;2*, *HvTIP2;1*, *HvTIP2;2* and *HvTIP2;3*) and the second group of two genes (*HvTIP3;1*, *HvTIP4;1*) that were up-regulated after the drought treatment. The transcriptional activity of only one gene (*HvTIP4;1*) was not affected by the drought stress (Fig 7). The highest reduction of the expression under drought stress was detected for the *HvTIP1;2* and *HvTIP2;1* genes, a 142- and 30-fold lower expression level in the drought-treated seedlings than in the control seedlings, respectively. For three other genes, *HvTIP1;1*, *HvTIP2;3* and *HvTIP2;2*, this decrease was much lower– 7-, 5- and 1.5-fold, compared to the control, respectively. The greatest change in transcriptional activity was observed for the *HvTIP3;1* gene, which showed more than a 5000-fold increase in expression in the leaves of the stress-treated seedlings. Interestingly, rehydration caused the return of the expression of many genes to the level that was observed under optimal moisture conditions or at least a change in this direction (Fig 7). For the *HvTIP1;1* and *HvTIP1;2* genes, the expression increased again, while for the *HvTIP3;1* and *HvTIP4;1* genes, it dropped to the control level. After the re-watering period, only three of the eight genes (*HvTIP2;3*, *HvTIP2;1* and *HvTIP2;*2) had a change in their expression level compared to the control growth. One of these genes–*HvTIP2;3* exhibited a further decrease in expression level after the significant decrease that was caused by the drought treatment and its expression after re-watering was reduced 59-fold lower compared to control. That was the only gene that showed a specific profile than all of the other *HvTIP* genes that were analysed in this study, i.e. changes in the opposite direction after re-watering. Based on the observations presented above, we conclude that HvTIP play an important function during the adaptation of barley to drought stress conditions.

## Discussion

Aquaporins are present in diverse living organisms including vertebrates, invertebrates, microorganisms and plants. They have a high degree of diversity and abundance in the plant kingdom. A subfamily of aquaporins–tonoplast intrinsic proteins (TIP) is implicated to be involved in the bidirectional flow of water and other substrates, primarily *via* the tonoplast. They are important for ensuring the appropriate turgor of individual cells. Plants, which are sessile organisms, need to continuously adjust their water status in response to changing environmental conditions and aquaporins play an important role in this process [32]. However, there is only limited knowledge about the role of the TIP proteins in barley that has been exposed to drought stress, which we address in this study.

Our results indicate that among the 11 *HvTIP* genes that were studied, *HvTIP2;3*, *HvTIP1;2* and *HvTIP1;1* were most abundantly expressed in the leaves of ten-day-old barley seedlings being grown under optimal water conditions. This may suggest that they play a broader role in this organ as well as during this developmental stage. Maintaining the water homeostasis during growth under optimal water conditions also requires their involvement [19]. According to Besee et al. [13], these three aquaporins are able to transport water; however, experiments by Ligaba et al. [15] did not confirm this ability for HvTIP2;3. Our results are in line with previous reports that have shown that TIP1;1 is the most widely distributed and abundantly expressed TIP isoform in different plant species and tissues [14, 16, 33]. Out of the 11 *HvTIP* genes that were studied, we did not detect the expression of three of them (*HvTIP3;2*, *HvTIP4;3* and *HvTIP5;1*) in barley seedlings that were grown under both optimal conditions and drought stress. Their expression may be tissue- or development-dependent. A previous study in barley indicated that the HvTIP3;2 isoform is engaged in the transport of only one substrate–hydrogen peroxide; however, no information is available about the substrate specificity of HvTIP4;3 and HvTIP5;1 [12]. The *HvTIP5;1* gene showed no expression in the leaf tissue of 16-day-old seedlings that had been cultivated in hydroponics, which is in line with our results [13]. The organ- and development-specificity of the TIP5;1 and TIP3;2 isoforms has also been proven in other species. The Arabidopsis *AtTIP5;1* gene was found to be expressed only in the sperm cell, stamen and pollen [33], while in *Eucalyptus* species, *EgTIP5;1* was the only gene from the TIP subfamily that appeared to be nonfunctional [34]. Another TIP isoform, *TIP3;2*, which was not expressed in the barley leaves in our study, had been highly specifically expressed in *A*. *thaliana* (only during senescense) and in *Oryza sativa* (in the mature seeds, spikelet and callus) [33, 35]. It should be noted that other *TIP* aquaporin genes were expressed at almost the same levels during each developmental stage in Arabidopsis [33].

Aquaporins are responsible for precisely regulating the movement of water and therefore may play a crucial role in the drought-stress response as well as in drought-stress tolerance [36]. In our study, the expression of all three *HvTIP* aquaporins that are involved in the transport of water–*HvTIP1;1*, *HvTIP1;2* and *HvTIP2;3* was down-regulated after ten days of severe drought treatment. This could lead to a decrease in the water permeability of membranes in order to avoid water loss and to minimize the water flow through the cell membranes to prevent the further loss of leaf turgor. A similar expression pattern has been shown for the seven genes in other studies. In *Festuca* species, the transcript level of the *TIP1;1* aquaporin decreased after 11 days of a water deficit [18]. In *Nicotiana glauca*, the *NgMIP2* and *NgMIP3* genes, which are homologous to TIP, were down-regulated under drought stress [19] and in Arabidopsis, the levels of *AtTIP1;1*, *AtTIP1;2*, *AtTIP2;1* and *AtTIP2;2* were down-regulated more than four-fold after 12 days of drought [16]. In contrast to these results, the expression level of *AtTIP1;1* and *AtTIP2;1* under drought treatment was up-regulated, but the time of the applied stress was only 24 hours [17]. We also observed a significant decrease in the expression level of *HvTIP2;1* and slightly smaller decrease for *HvTIP2;2*. There is no experimental evidence for water transport by HvTIP2;2 in barley and HvTIP2;1 in the study of Ligaba et al. [15] showed no water permeability. In spite of this data, the down-regulation of its expression might suggest that *HvTIP2;1* could be involved in water transport in barley that is under drought stress.

After 14 days of re-watering, the expression level of *HvTIP1;1* and *HvTIP1;*2 (whose transcriptional activity was down-regulated under drought stress), returned to the level that was present in the non-stressed plants, while the expression of *HvTIP2;1* and *HvTIP2;2* increased but did not reach the level of the control plants. The expression of only one gene–*HvTIP2;3* dropped further after re-watering compared to the plants under drought stress. This may indicate a different function of *HvTIP2;3* in response to stress. Because the transcript of the *HvTIP2;3* gene was the most abundant in the barley leaves under the optimal moisture conditions, its regulation should have an significant impact on the plant response at the physiological level. In Arabidopsis, 26 h after re-hydration, the expression of the *AtTIP1;1*, *AtTIP1;2*, *AtTIP2;1* and *AtTIP2;2* genes in the drought-stressed plants were back at the same levels as in the control plants [16].

Among all of the investigated aquaporin genes, two, *HvTIP3;1* and *HvTIP4;1*, were up-regulated. *HvTIP3;1* expressed the highest increase in its activity (ca. 5000-fold) under drought stress, thus indicating the important role of the encoded protein in the response to drought. This aquaporin might help plants to adapt or tolerate stress condition. This isoform is predicated to transport hydrogen peroxide [12], which is a close chemical analogue of water. H_2_O_2_ is not only a toxic metabolic by-product but also a significant intermediate [37]. The efficient transmembrane diffusion of H_2_O_2_ requires aquaporin activity, which makes these channels important players in the redox signalling network [38] and in H_2_O_2_ detoxification [39–41]. Only one other gene, *HvTIP4;1*, was up-regulated in response to drought, although to a much lower degree compared to *HvTIP3;1*. There is no prediction of any non-aqua substrate transport for this aquaporin [12]. Interestingly, after re-watering, the expression level of both *HvTIP3;1* and *HvTIP4;1* returned to the level in non-stressed plants.

An analysis of the putative *cis*-regulatory elements that were present in the promoter sequences of aquaporin genes belonging to TIP subfamily led to the conclusion that their expression may be regulated in response to hormones such as abscisic acid (ABA), methyl jasmonate (MeJA), auxin and gibberellin (GA). The presence of ABRE in the investigated promoters suggests a possible role of ABA in the control of *HvTIP* expression as has already been reported for some *TIP* genes in barley. The expression of the *HvTIP3;1* and *HvTIP1;2* genes increased in the aleurone cells after ABA treatment and was strongly repressed by gibberellin [42]. After ABA treatment, the *HvTIP1;2* gene had a different expression pattern in barley shoots and roots, down- and up-regulation, respectively [15]. Many of the abiotic stress-inducible genes contain two *cis*-acting elements: a dehydration-responsive element (DRE, TACCGACAT; DRE-core A/GCCGAC) and an ABA-responsive element (ABRE, ACGTGG/TC) in their promoter region [43]. In the *HvTIP*, these two *cis*-acting elements are present in the promoters of *HvTIP1;1*, *HvTIP2;2*, *HvTIP2;3*, *HvTIP4;1*, *HvTIP4;3* and *HvTIP5;1*. The other two well-represented regulatory elements that were found in all of the promoters that were investigated here were the CGTCA- and TGACG-motifs. Both of these elements are implicated in the plant response to methyl jasmonate (MeJA), which is a signalling molecule that is involved in the stress response [44]. The variety and richness of the identified *cis*-regulatory elements in the *HvTIP* promoters indicate their engagement in the response to abiotic stresses, which was confirmed in the presented study. The lack of expression modulation after drought treatment was found for only one gene (*HvTIP4;2*) and may be explained by the involvement of this isoform in the stress response in a different organ or during a different developmental stage.

In this study we have identified drought-responsive tonoplast aquaporins in barley, which is one the fourth cereal species in terms of production and cultivation area worldwide. We have indicated the TIP isoforms, whose expression was highly induced by drought, but further studies are needed to reveal the relationship between the action of a particular aquaporin and drought tolerance. Due to the climatic crisis and changing environmental conditions around the world, breeding of drought tolerant cultivars is a major challenge of the modern agriculture. The identified genes can be used for more detail studies that enable their use as targets for gene manipulation towards receiving drought tolerant cereals.

## Supporting information

S1 Table Primers used in qRT-PCR. (DOCX)

## Abbreviations

ABA: abscisic acid
APQ: aquaporins
AWD: adaptation to water deficit
CG: control growth
DAS: days after sowing
DRE: dehydration-responsive element
DS: drought stress
GA: gibberellins
LTR: low temperature-responsive elements
MBS: MYB binding site involved in drought-inducibility
MeJA: methyl jasmonate
MIP: membrane intrinsic proteins
NPA: asparagine-proline-alanine
RW: re-watering phase
RWC: relative water content
TIP: tonoplast intrinsic proteins
