# Supplementary material for: Drought stress and re-watering affect the abundance of TIP aquaporin transcripts in barley
Source: PLoS One. 2019 Dec 17;14(12):e0226423. doi: 10.1371/journal.pone.0226423 (PMC6917287; doi:10.1371/journal.pone.0226423)
Supplement: S1 Table — (DOCX) [file pone.0226423.s001.docx]

| **Primer name** | **Forward sequence** | **Reverse sequence** | **Annealing temperature (°C)** |
| --- | --- | --- | --- |
| ADP | 5’-CGTGACGCTGTGTTGCTTGT-3’ | 5’-CCGCATTCATCGCATTAGG-3’ | 58 |
| HvTIP1;1_1 | 5’-CTTCCTCCTCCGCTTCTCC-3’ | 5’-ATGACGATCTCCAGGACCAC-3’ | 58 |
| HvTIP1;2_2 | 5’-CTCCTCAAGATCGCCACTG-3’ | 5’-ATGACGATCTCGAACACCAC-3’ | 58 |
| HvTIP2;1_2 | 5’-GCTGGCAACTGGGTCTACTG-3’ | 5’-CTGGTAGGACGCGATGAAC-3’ | 58 |
| HvTIP2;2_6 | 5’-CACCCTCCTCTTCGTGTTCC-3’ | 5’-CGACGAAGAGGGCGAAGG-3’ | 58 |
| HvTIP2;3_2 | 5’-CGGCGACTTCACCAATATCT-3’ | 5’-GGTGTGGTTGTCGCACAT-3’ | 58 |
| HvTIP3;1_2 | 5’-AGGCACCACTGGGTCTACTG-3’ | 5’-CTCTGTAGACGGGATGACGA-3’ | 58 |
| HvTIP4;1_1 | 5’-CCTCACCTTCTCCCTCCTCT-3’ | 5’-GTGTTGGCACCGACAATAAG-3’ | 58 |
| HvTIP4;2_3 | 5’-TTCGAGGGCTGGCTTGATTT-3’ | 5’-CCTCAACGTCCTCAACACGA-3’ | 59 |
